# The Role of Exercise to Reduce the Impact of Diabetes in the Seminal Quality: A Systematic Review

**DOI:** 10.3390/medicina57020159

**Published:** 2021-02-10

**Authors:** Ana Myriam Lavín-Pérez, Daniel Collado-Mateo, Santos Villafaina, Violeta Calle-Guisado

**Affiliations:** 1 Centre for Sport Studies, Rey Juan Carlos University, Fuenlabrada, 28943 Madrid, Spain; am.lavin.2018@alumnos.urjc.es (A.M.L.-P.); daniel.collado@urjc.es (D.C.-M.); 2 GO fitLAB, Ingesport, 28003 Madrid, Spain; 3 Physical Activity and Quality of Life Research Group (AFYCAV), Faculty of Sport Sciences, University of Extremadura, 10004 Cáceres, Spain; 4 Department of Specific Didactics, Faculty of Human Sciences and Education, Experimental Sciences Area, University of Zaragoza, 50009 Zaragoza, Spain; violetacg@unex.es

**Keywords:** diabetes, physical exercise, sperm quality, fertility, human, animals

## Abstract

*Background and Objectives*: One of the most relevant consequences of diabetes mellitus is the temporal or complete infertility which can happen in young individuals. Therefore, the current systematic review aimed to investigate the effects of exercise to reduce the impact of Type 2 Diabetes Mellitus (T2DM) in seminal quality and related parameters. *Materials and Methods*: A systematic search was conducted in Pubmed and Web of Science databases following the Preferred Reporting Items for Systematic Reviews and Meta-Analyses Guidelines (PRISMA). The inclusion criteria were: (1) the study included at least one experimental and one comparison group, (2) the sample of the study was comprised of humans or animals with diabetes mellitus, (3) an intervention based on physical exercise was conducted, and (4) the study reported variables related to the seminal quality. *Results*: A total of 115 articles were identified. However, only six accomplished the inclusion and exclusion criteria. This systematic review includes a sample size of 260 participants (180 rats and 80 humans). Intervention ranged from 6 to 14 weeks, with 3–6 days per week. All interventions performed endurance training (50–70% VO_2_max or maximum heart rate). Physical exercise increased sperm count, motility, and morphology, as well as improved testosterone, Luteinizing Hormone (LH), and Follicle Stimulating Hormone (FSH) levels. Moreover, physical exercise intervention reduced the percentages of sperms with negative Tubular Differentiation Index (TDI) and Spermiogenesis Index (SPI), DNA fragmentation, and also ameliorated the diabetes-induced apoptosis and improved sperm apoptosis index. *Conclusions*: Physical exercise could ameliorate diabetic pathological effects on sperm quality and related parameters that cause infertility or subfertility conditions. However, further homogeneous studies are needed to confirm these findings.

## 1. Introduction

The current diabetes mellitus prevalence is estimated in an uncertain interval from 340 to 536 million people worldwide. Type 2 Diabetes mellitus (T2DM) approximately make up 85–95% of diagnosed cases of diabetes. The International Federation of Diabetes suggests that, without prevention programs, the prevalence will rise to 10.9% of the global population in 2045 (700 million), making it one of the leading health problems with a high sanitary cost [1]. In addition, the working-age population from 20 to 69 years old accounts for more than 75% of diabetes cases in the world [2]. Diabetes mellitus has been associated with pathological consequences in the global organism due to the inability to regulate glucose metabolism [3]. One of the most important is the temporal or complete infertility that can occur in young individuals of reproductive age [4].

There is a global crisis in male reproductive health due to a progressive decrease in the quality values of human semen and increasing male reproductive system abnormalities [5]. In animals, T2DM may impair male fertility at multiple levels, both dysregulation spermatogenesis endocrine control or by damaging penile erection and ejaculation [6,7]. In humans, the prevalence of subinfertility in people with diabetes mellitus is well known, but the pathophysiological mechanisms of damage are different in type 1 diabetes mellitus and T2DM. In patients with T2DM, the subquality of semen may be caused by the increased oxidative stress [8]. There is strong evidence that physical activity prevents the risk of T2DM and can ameliorate the symptoms in patients with this pathology due to the antioxidant effect of the regulation of glucose blood levels, and improve insulin sensitivity [9]. It is also reported that physical activity has beneficial effects on sperm quality of sedentary obese adults [10] and healthy subjects [11], as well as on oxidative stress testicular function [12] and reproductive programming in the offspring [13]. However, there is no systematic review of the effect of physical exercise on semen quality and other related parameters in patients with diabetes mellitus. Therefore, the current systematic review aimed to investigate the effects of exercise to reduce the impact of T2DM in seminal quality and related parameters.

## 2. Materials and Methods

### 2.1. Data Sources and Searches

The following database resources were used to collect the articles: PubMed (MEDLINE) and Web of Science (including Current Contents Connect, Derwent Innovations Index, Korean Journal Database, Medline, Russian Science Citation Index, SciELO Citation Index). The search terms employed were: (“diabetes” or “glucose”) and (“exercise” or “physical activity) and (“sperm” or “semen” or “seminal”). Duplicated studies were manually excluded. The search procedure ended in May 2020.

The articles were included in the review according to the following criteria: (1) the study included at least one experimental and one comparison group, (2) the sample of the study was comprised of humans or animals with diabetes, (3) there was an intervention based on physical exercise, (4) the study reported variables related to the seminal quality.

**Figure 1:**
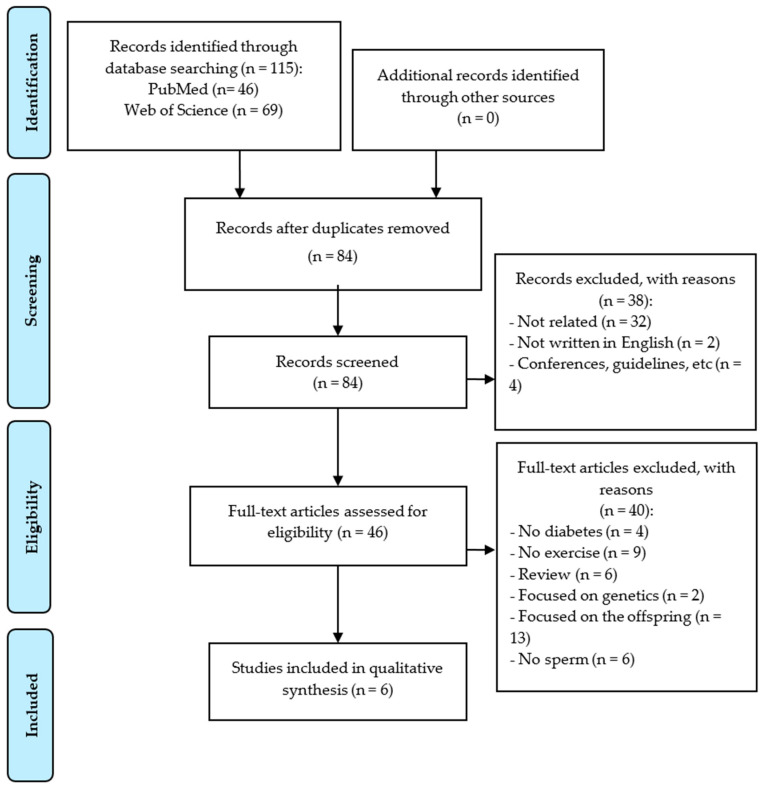
Diagram flow of the systematic review.

Moreover, the following exclusion criteria were set: (a) articles not written in Spanish or English, (b) articles with one single group, and (c) articles focused on genetic variables. The selection procedure is shown in Figure 1. It was performed by one of the authors and checked by another one.

### 2.2. Risk of Bias

The Evidence Project tool was used to assess the risk of bias [14] (see Table 1). This tool can be used to rate articles with different designs, including randomized and non-randomized controlled or uncontrolled trials. This scale involves three dimensions, including: (1) the study design, with the items “cohort”, “control or comparison group” and pre-post intervention data; (2) the participants’ representativeness, with the items “random assignment of participants to the intervention”, “random selection of participants for assessment”, and “follow-up rate of 80% or more”; and (3) the equivalence of comparison groups, including the items “comparison groups equivalent on sociodemographics”, and “comparison groups equivalent at baseline on outcome measures”.

### 2.3. Data Extraction

The extraction process was conducted following the PICOS approach collected in PRISMA guidelines [15]. PICOS is the acronym for participants, intervention, comparisons, results, and study design. Thus, regarding participants, Table 2 includes the main sample characteristics (number of participants in each study group, age, and weight) as well as the study design. Table 3 shows the interventions conducted for each study, with a special focus on both physical exercise and other interventions conducted. The experimental characteristics reported in Table 3 include exercise frequency, session duration, type of exercise performed, and workload progression. The outcome assessment (including the instruments used and the procedure carried out) and results are summarized in Table 4. Furthermore, results in other variables can be observed in the Table A1 The main variables included in Table 4 are related to the sperm analysis, involving cell count, viability, morphology, motility, and the apoptosis index. Other variables such as the Luteinizing Hormone (LH), Follicle Stimulating Hormone (FSH), testosterone, and adiponectin levels were included in the Table A1. Both between-group and within-group comparisons were extracted from the original articles to provide information about the exercise effects compared to baseline or a control group under diverse conditions (non-diabetic, sedentary, etc.).

## 3. Results

### 3.1. Study Selection

Figure 1 shows the article selection and the main reasons for exclusion. A total of 115 articles were identified (46 in PubMed and 69 in Web of Science). After removing the duplicated ones, a total of 84 studies were examined. Thirty-eight investigations were excluded since they have not been related to the current topic (n = 32), have not been written in English (n = 2), or been guidelines or conference abstracts (n = 4). A full-text analysis of 46 articles was then conducted, and only six accomplished the inclusion and exclusion criteria. The eliminated articles were: six reviews, four interventions without diabetic participants, nine without an exercise program, and six without sperm assessment, as well as thirteen articles that were focused on offspring and two on genetics.

### 3.2. Risk of Bias

Table 1 shows the score of each article according to the Evidence Project tool. Results ranged from 3/8 to 7/8. The studies were divided according to the sample characteristics into: (a) humans and (b) animals. It must be noted that some items such as “random assignment of participants to the intervention” or “random selection of participants for assessment” could lead to some errors when assessing the risk of bias. Given that the authors did not report whether the assignment was random or not, we have considered that this assignment could not be randomized. Regarding the “random selection of participants”, we have considered that the animals were randomly selected. Furthermore, the lack of baseline measures in two of the studies [16,17] made it impossible to judge if there were differences at baseline, so items 7 and 8 could have been scored as “N/A”.

Regarding the studies with humans, one of the studies included a sample comprised of diabetic and non-diabetic people performing the same exercise intervention (single-session intervention) [18]. Thus, the comparison groups had no equivalence, whereas all the articles left include one or more groups to compare the principal variables.

**Table 1:** Risk of bias assessment using the Evidence Project tool.

Study	Study Design			Participant Representativeness			Equivalence of Comparison Groups		Total Score
	Item 1	Item 2	Item 3	Item 4	Item 5	Item 6	Item 7	Item 8	
Humans									
Rosety Rodríguez 2014 [19]	Yes	Yes	Yes	Yes	No	Yes	Yes	Yes	7/8
Murray 1988 [18]	Yes	No	Yes	No	No	Yes	No	No	3/8
Rats									
Samadian 2019a [16]	No	Yes	No	No	Yes	Yes	N/A	N/A	3/6
Samadian 2019b [17]	No	Yes	No	No	Yes	Yes	N/A	N/A	3/6
Parastesh 2019a [20]	Yes	Yes	No	No	Yes	Yes	Yes	N/A	5/7
Parastesh 2019b [21]	Yes	Yes	No	No	Yes	Yes	Yes	N/A	5/7

Items of the Evidence Project tool: (1) cohort, (2) control or comparison group, (3) pre-post intervention data, (4) random assignment of participants to the intervention, (5) random selection of participants for assessment, (6) follow-up rate of 80% or more, (7) comparison groups equivalent on sociodemographics, and (8) comparison groups equivalent at baseline on outcome measures.

### 3.3. Study Characteristics

The current systematic review includes a sample size of 260 participants (180 rats and 80 humans) divided into the following groups: healthy control group (Con) (n = 40), diabetic control group (Dia) (n = 60), diabetic and insulin injection intervention (DiaIn) (n = 18), healthy control group participating in an exercise intervention (Ex) (n = 30), diabetic group participating in an exercise intervention (DiaEx) (n = 848), and a diabetic group with regulated insulin injection and participating in an exercise intervention (DiaInEx) (n = 28). The diabetic groups incorporated T2DM [19,20,21] and type I diabetes mellitus [16,17,18]. Mice aged eight weeks [20,21] and aged two months [16,17] and their weights ranged from 180 g to 250 g. In the human studies included, the participants had a mean age of 36.09 years old and a 30% fat mass [19] or a body mass index of 22.8 kg/m^2^ [18] (shown in Table 2).

### 3.4. Interventions and Comparison Groups

The articles collected reported an exercise intervention, and in those with mice as subjects, also distinguished an insulin-dependent intervention with and without exercise. The exercise programs’ mean length was 9.2 ± 3.35 weeks (from 6 to 14), although one study investigated the effects of acute exercise planning a unique session [18]. As Table 3 shows, participants exercised three [19], five [16,17,21], and six [20] days/week, and the sessions had an approximated mean duration of 43.2 min (including the acute exercise session). The type of exercise performed in all the interventions was endurance training, concretely running [16,17,19,20,21], and cycling [18]. The intensity of the aerobic exercise carried out was moderate: 50% VO_2_max [18], 65% VO_2_max (18 m/min) [16,17], 55–70% HRpeak [19] and 27 m/min [20,21]. Participants followed a training progression during the exercise programs. This adjustment was made by increasing the running speed (from 9 m/min to 18 m/min) [16,17] and/or raising the session duration (from 15 min to 30 min [16,17] or incrementing 2 min of duration each session until 60 min were reached) or increasing the intensity with a 2.5% HRpeak enhancement every two weeks [19]. Resistance training was only included in Parastesh et al. (2019) study where mice climbed 12 times (three sets of four repetitions) a 1 m-long-laded carrying weight in their tail (increasing the weight from 30% to 200% of the animal body weight) [21]. The strength exercise was performed by the specific resistance training group (DiaInExR) and the combined exercise group (DiaInExC) [21].

As for the insulin ejection interventions, the articles incorporated its consumption with and without participating in an exercise program [16,17]. The doses utilized were five units of isophane insulin (NPH), in the acute exercise study [18], and 0.9 International Unit (IU)/100 g [16,17].

### 3.5. Outcome Measures

The effects of exercise on sperm quality are summarized in Table 4.

#### 3.5.1. Sperm Count and Motility

**Figure 2:**
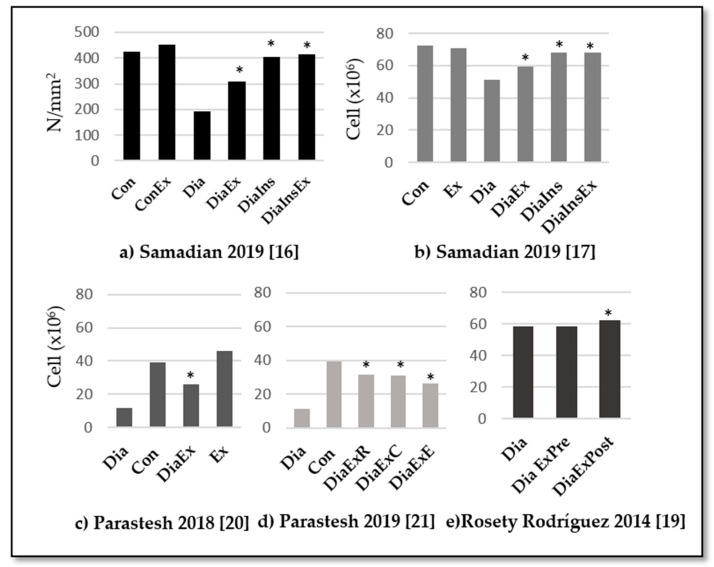
Summary of results for sperm count. Con: control group, Dia: diabetic group, DiaIns: diabetic and insulin administration group, DiaEx: diabetic and exercise intervention group, DiaInEx: diabetic group with insulin administration and exercise intervention, DiaExE: diabetic and endurance exercise group, DiaExR: diabetic and resistance exercise group, DiaExC: diabetic and combined exercise group. DiaExpre: diabetic group baseline values, DiaExpost: diabetic group post-exercise intervention. *: *p* < 0.05 between experimental group (exercise, insulin, or both) and diabetic control group. Main comparisons: (**a**,**b**) Con = Ex = DiaIn = DiaInEx > DiaEx > Dia. *p* < 0.05. (**c**) Dia (11.75 ± 5.7) < DiaEx (26 ± 13.2) < Con (39.3 ± 13) = Ex (46.2 ± 3.3)]. *p* < 0.05. (**d**) DiaExR (31.7 ± 10) = DiaExC (31 ± 5.7) > Dia (11.7 ± 5.7); DiaExE: 26 ± 13.2 = Dia (11.7 ± 5.7). *p* < 0.05. (**e**) DiaEx (pre, 58.6 ± 5.9) < DiaEx (post, 62.2 ± 6.3) > Dia (post, 58.2 ± 6.5). *p* < 0.05.

All studies evaluated the sperm count and supported physical exercise benefits to improve cell count, volume, and/or concentration in diabetic samples. In rats, Samadian et al. [16,17] and Parastesh [20,21] observed how exercise reduced the effect of diabetes mellitus on sperm count (see Figure 2), and those results were even better when it was combined with insulin treatment (*p* < 0.05) [16,17] or when the physical exercise was not limited to endurance training but included resistance training [21]. In humans, Murray et al. [18] observed a non-significantly lower seminal volume and density in the participants with diabetes mellitus, while Rosety-Rodríguez et al. [19] found significant changes after an exercise program in sperm concentration (*p* = 0.029) but not in volume.

**Figure 3:**
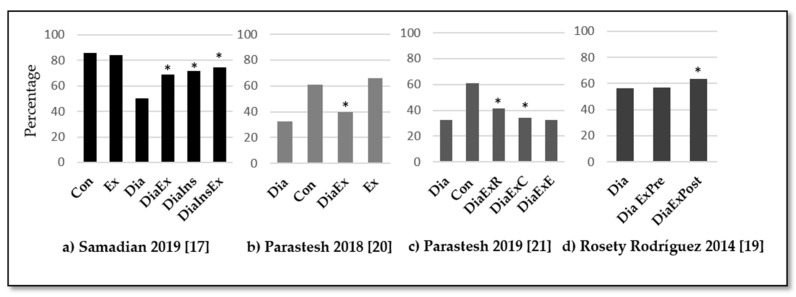
Summary of results for sperm motility percentage. Con: control group, Dia: diabetic group, DiaIns: diabetic and insulin administration group, DiaEx: diabetic and exercise intervention group, DiaInEx: diabetic group with insulin administration and exercise intervention, DiaExE: diabetic and endurance exercise group, DiaExR: diabetic and resistance exercise group, DiaExC: diabetic and combined exercise group. DiaExpre: diabetic group baseline values, DiaExpost: diabetic group post-exercise intervention. *: *p* < 0.05 between experimental group (exercise, insulin, or both) and diabetic control group. Main comparisons: (**a**) Con = Ex > DiaIn = DiaInEx = DiaEx > Dia. *p* < 0.05. (**b**) Ex (66.1 ± 4.1) > Con (60.8 ± 6.5) > [DiaEx (40 ± 6.5) = Dia (32.5 ± 1.1). *p* < 0.05. (**c**) DiaExR (41.4 ± 4.5%) = DiaExC (34.4 ± 2.6%) > Dia (32.5 ± 1.1%). *p* < 0.05. (**d**) DiaEx (pre, 57.1 ± 5.4) < DiaEx (post, 63.5 ± 5.8) > Dia (post, 56.3 ± 6.0). *p* < 0.05.

In diabetic rats, physical exercise increased the motility combined or not to the insulin treatment (*p <* 0.05) [17,20,21]. However, endurance training was not enough to significantly improve sperm motility, while resistance training or a combination of endurance and resistance training were able to achieve that effect (*p* < 0.05) [20,21]. In humans, Murray et al. [18] detected a 13% reduction of motility among diabetic patients compared to healthy subjects. Furthermore, Rosety-Rodríguez et al. [19] observed a significant increment of 10% in motility after an exercise intervention (*p* = 0.006). These results are summarized in Figure 3.

#### 3.5.2. Sperm Morphology

Studies observed a difference between diabetic and non-diabetic samples in rats, but there was no clear evidence of the benefits of physical exercise on sperm morphology. On the other hand, in humans, Rosety-Rodríguez et al. found significant improvements in this variable *(p* = 0.018).

#### 3.5.3. Other Variables

Significant improvements have also been observed in other variables such as the apoptosis index, the impaired chromatin condensation, and DNA fragmentation, with further benefits when combined with insulin treatment for all those three variables [16,17].

The Table A1 includes the results for many other variables. The Tubular Differentiation Index (TDI) and the Spermiogenesis Index (SPI) were improved after a physical exercise program, especially combined with insulin treatment [16,17]. Regarding hormonal levels, testosterone was significantly higher in diabetic rats combined or not with insulin treatment [17] (*p* < 0.05). This is in line with the findings by Parastesh et al. [21], who observed that the testosterone levels of diabetic samples after exercise were even higher than those from the healthy samples, which also happens for the LH and FSH levels.

## 4. Discussion

This systematic review aimed to evaluate the current scientific evidence about the role of physical exercise to reduce the potential effect of diabetes on sperm quality. Six studies were included, and results showed that regular physical exercise for six weeks or more could increase sperm count, motility, and morphology both in humans (2/6 articles) and rats (4/6 articles), as well as to improve the testosterone, LH, and FSH levels in rats. Also, exercise can reduce the percentages of sperms with negative TDI and SPI, DNA fragmentation, and also ameliorate the diabetes-induced apoptosis and improve sperm apoptosis index in animal models.

A previous meta-analysis, including more than 1000 patients with diabetes mellitus, showed the harmful effects of diabetes in functional sperm characteristics [24]. T2DM is often a complication of obesity and may have a large impact on male fertility through some associated consequences such as hyperinsulinemia, hyperleptinemia, chronic inflammation, and oxidative stress [25]. In this regard, increasing the body mass index is associated with lower ejaculate volume, sperm density, motility, or morphology, as well as with a lower testosterone level [26]. In order to reduce those negative effects and improve fertility, lifestyle modifications must be applied. Physical exercise is one of the most important strategies to reduce and prevent obesity and other non-communicable diseases, improving health status and quality of life. The effects of physical exercise on healthy male reproductive outcomes have been investigated before, showing improvements in hormonal levels, semen concentration, and motility [12,27]. The current systematic review results are in line with those previous studies, showing that physical exercise can reduce the impact of diabetes in sperm quality, hormonal levels, and even leading them to similar or higher values than those observed in healthy controls. Also, other sperm quality parameters related to DNA fragmentation, spermiogenesis, germ cellular differentiation, and apoptosis index have been considered and results show an improvement of these values with exercise.

Although the potential benefits of physical exercise have been largely studied in the scientific literature, there are still some controversial approaches regarding not only the benefits of regular exercise and which variables can be improved but also which type of physical exercise is the best choice to improve male fertility. Two recent reviews showed how the effects of physical exercise depend on the type of exercise and the profile of the participant [27,28]. They observed that high-intensity training with increased loads might negatively affect sperm quality by impairing semen parameters and fertilization potential. On the other hand, they also found that in recreational athletes, the effect was neutral or even positive. In line with those results, a large randomized controlled trial involving 433 infertile men training at 70–85% of their maximal oxygen consumption showed that high-intensity exercise might reduce the inflammatory biomarkers, oxidative stress, and antioxidants, as well as improve the semen parameters and the pregnancy rate [29]. The results presented in the current systematic review are consistent with those findings. In this regard, the studies by Parastesh et al. [20,21] in rats suggest that resistance training (climbing a ladder with a load) and the combination of endurance plus resistance training may lead to further benefits than endurance training alone. However, none of the studies included in this systematic review conducted training at a high intensity (always lower than 70% of the maximum oxygen consumption). Therefore, future studies should evaluate the effects of exercising at different intensities and, specifically, the effects of high-intensity exercise on the sperm quality of people with diabetes mellitus.

Diabetes mellitus induces cell apoptosis [30] that can cause adverse effects on sperm quality. The protective effect of exercise on a diabetes apoptosis-induced pathway has also been studied in two studies performed in rats included in this review [16,17], showing an up-regulation of anti-apoptotic genetic factors. However, more clinical trials in the same line are required to consider moderate exercise as a genetic regulator of diabetes apoptosis effect.

Some limitations must be considered in the current study. The first one is related to the low number of studies, which impeded the calculation of a meta-analysis. Secondly, there was a very high heterogeneity of samples, measures, and exercise protocols, which make it difficult to extract clear conclusions. The third limitation is that the majority of the studies (4/6) are performed with animal models (rats), so the extrapolation of the results to humans may be limited. Finally, a historical cohort study is necessary to discuss the observed effects on pregnancy and fertility rates that continue to be unclear.

## 5. Conclusions

Physical exercise could ameliorate diabetic pathological effects on sperm quality and related parameters that cause infertility or subinfertility conditions. Results showed that regular physical exercise for six weeks or more could increase sperm count, motility, and morphology, as well as to improve the testosterone, LH, and FSH levels. Moreover, exercise could be able to reduce the percentages of sperms with negative TDI and SPI, DNA fragmentation, and also ameliorate the diabetes-induced apoptosis and improve sperm apoptosis index. However, further homogeneous studies are needed to confirm these findings, especially in human subjects.

## Figures and Tables

**Table 2:** Baseline characteristics of the participants included in the systematic review.

Study	Study Design	Group: Sample Size	Age	Weight/Body Composition
Samadian 2019a [16]	Controlled trial	Sedentary Con: n = 6 Sedentary Dia: n = 6 Sedentary DiaIn: n = 6 Ex: n = 6 DiaEx: n = 6 DiaInEx: n = 6	Two-month-old male Wistar rats	180–220 g
Samadian 2019b [17]	Controlled trial	Sedentary Con: n = 6 Dia: n = 6 DiaIn: n = 6 Ex: n = 6 DiaEx: n = 6 DiaInEx: n = 6	Two-month-old male Wistar rats	180–220 g
Parastesh 2019a [20]	Controlled trial	Con: n = 12 Dia: n = 12 DiaIn: n = 12 Ex: n = 12 DiaEx: n = 12 DiaInEx: n = 12	8-wk old Sprague Dawley rats	200–250 g
Parastesh 2019b [21]	Controlled trial	Con: n = 12 Dia: n = 12 DiaExE: n = 12 DiaExR: n = 12 DiaExC: n = 12	8-wk old Sprague Dawley rats	200–250 g
Rosety Rodríguez 2014 [19]	Randomized controlled trial	Dia. n = 30 DiaEx: n = 30	Dia: 35.7 ± 4.0 years DiaEx: 36.2 ± 3.5 years	Dia: 29.7 ± 2.6% Fat mass DiaEx: 30.3 ± 2.8% Fat mass
Murray 1988 [18]	Controlled trial	Ex: n = 10 DiaInEx: n = 8	Ex: 26 ± 1.7 years DiaInEx: 23 ± 0.8 years	Ex: 22.4 ± 1.24 body mass index DiaInEx: 23.2 ± 1.50 body mass index

Con: control group; Dia: diabetic control group; DiaIn: insulin administration group; DiaEx: diabetic and exercise intervention group; DiaInEx: diabetic group with insulin administration and exercise intervention; DiaExE: diabetic and endurance exercise intervention group; DiaExR: diabetic and resistance exercise intervention group; DiaExC: diabetic and combined exercise intervention group.

**Table 3:** Characteristics of the interventions included in the systematic review.

Study	Group	Intervention Duration	Frequency	Session Duration	Description	Progression	Additional Information
Samadian 2019 [16]	Con	6 weeks	None	None	None	None	
	Dia		None	None	None	None	
	DiaIn		None	None	None	None	Insulin: 0.9 IU.100/g once a day for 6 weeks
	Ex		5 days/wk	25 min (mean)	5 min warm-up and cool-down Running at 18 m/min at 5% of inclination and 65% VO_2max_ from 9 to 18 min	Speed: from 9 m/min to 18 m/min Duration: from 15 min to 30 min	
	DiaEx						
	DiaInEx						Insulin: 0.9 IU.100 g^−1^ once a day for 6 weeks
Samadian 2019 [17]	Con	6 weeks	None	None	None	None	
	Dia		None	None	None	None	
	DiaIn		None	None	None	None	Insulin: 0.9 IU.100 g^−1^ once a day for 6 weeks
	Ex		5 days/wk	25 min (mean)	5 min warm-up and cool-down Running at 18 m/min at 5% of inclination and 65% VO_2max_ from 9 to 18 min	Speed: from 9 m/min to 18 m/min Duration: from 15 min to 30 min	
	DiaEx						
	DiaInEx						Insulin: 0.9 IU.100 g^−1^ once a day for 6 weeks
Parastesh 2019 [20]	Con	10 weeks	None	None	None	None	
	Dia		None	None	None	None	
	Ex		6 days/wk	52.1 min (mean)	5 min warm-up and cool-down (16 m/min) Running at 27 m/min and 0º inclination	Adaptation wk: walking 8 m/min Duration from 1st wk to 4th wk (2 min/session) until 60 min and maintain	
	DiaEx						
Parastesh 2019 [21]	Con	10 weeks	None	None	None	None	
	Dia		None	None	None	None	
	DiaExE		5 days/wk	52.1 min (mean)	5 min warm-up and cool-down (16 m/min) Running at 27 m/min and 0º inclination	Adaptation wk: walking 8 m/min Duration from 1st wk to 4th wk (2 min/session) until 60 min and maintain	
	DiaExR		5 days/wk	Not reported	1 m climbing a long ladder at 90º with a weight in animals’ tails 3 sets (2 min rest between) of 4 repetitions (10 s rest between)	Adaptation 1st wk Weight: 30% animal weight in 2nd wk and increase until 200% in 10th wk	
	DiaExC		5 days/wk	Alternatively: DiaExE protocol and DiaExR			
Rosety Rodríguez 2014 [19]	Dia	14 weeks	None	None	None	None	
	DiaEx		3 days/wk	~60 min	10–15 min warm-up 40 min treadmill at an intensity 55–70% HR_peak_ 5–10 min cool-down	Intensity: 2.5% HRpeak/2 wks	
Murray 1988 [18]	Ex	Acute session		45 min	Cycling at 50% VO_2max_	Workload: automatically readjust during the test.	
	DiaInEx						Diabetic: 5 units of NPH intermediate-acting insulin subcutaneously in the left hip 30 min before exercise

Con: control group, Dia: diabetic group, DiaIn: insulin administration group, DiaEx: diabetic and exercise intervention group, DiaInEx: diabetic group with insulin administration and exercise intervention, DiaExE: diabetic and endurance exercise intervention group, DiaExR: diabetic and resistance exercise intervention group, DiaExC: diabetic and combined exercise intervention group, VO_2_max: maximum oxygen consumption, HRmax: maximum heart rate.

**Table 4:** Evaluation procedure and results of sperm quality parameters.

Study	Variable	Measurement Procedure	Results
Samadian 2019 [16]	Cell count	Analyses by light microscopic	Figure 2
	Apoptosis index	Evaluated by TUNEL (Terminal deoxynucleotidyl transferase enzyme-mediated dUTP nick end labeling) staining kit (In situ cell death detection kit, POD, Roche; Cat, Germany).	(Con = Ex = DiaIn = DiaInEx) < DiaEx < Dia
Samadian 2019 [17]	Quality Sperm: Count	Incubation of tissues in heat transfer fluid (HTF) medium, 45 min at 37 °C in 5% CO2. Dilution of 1:50 and observation with light microscope stereo zoom microscope. Count based on standard Neobar slide method.	Figure 2
	Quality Sperm: Motility	Incubation of tissues in HTF medium, 45 min at 37 °C in 5% CO2. Dilution of 1:50 50 and observation with light microscope stereo zoom microscope.	Figure 3
	Quality Sperm: Impaired chromatin condensation	Aniline blue staining. The spermatozoa with unstained nuclei were considered as mature (representing protamine content), while the spermatozoa with stained nuclei (representing histone content) were marked as immature [22].	(Con = Ex) < (DiaIn = DiaInEx = DiaEx) < Dia
	Quality Sperm: DNA Fragmentation	Acridine orange Staining and evaluation by fluorescence microscope (Zeiss Company). Spermatozoa with green DNA content were marked as spermatozoa with double-stranded DNA, while the spermatozoa with yellowish and/or reddish fluorescent DNA content were considered as spermatozoa with DNA fragmentation.	(Con = Ex) < (DiaIn = DiaInEx = DiaEx) < Dia
Parastesh 2019 [20]	Sperm count (10^6^)	Dissected epididymis was transferred into a 5 cc (DMEM) medium. Sperm swim out into the medium. One mL of the solution was diluted with 9 mL formaldehyde fixative. Neubauer chamber and manually counted under a microscope.	Figure 2
	Sperm viability	Eosin-nigrosine staining was used to evaluate sperm viability according to WHO protocol [23].	Ex (87.8 ± 2.9%) > Con (77.5 ± 4.6%) > DiaEx (41.7 ± 7.2%) > Dia (29.7 ± 16.2%)
	Sperm morphology	Papanicolaou stain according to WHO protocol [23].	Día (85.2 ± 7.5) < Con (95.4 ± 1.3); Ex (90.4 ± 9.1) > (Dia (85.2 ± 7.5) = DiaEx (88 ± 8.8))
	Sperm motility	WHO protocol [23]. Ten µl of the sperm suspension was located on a microscope slide.	Figure 3
Parastesh 2019 [21]	Sperm count (10^6^)	Dissected epididymis was transferred into a 5 cc (DMEM) medium. Sperm swim out into the medium. 1 mL of the solution was diluted with 9 mL formaldehyde fixative. Neubauer chamber and manually counted under a microscope.	Figure 2
	Sperm viability	Eosin-nigrosine staining was used to evaluate sperm viability according to WHO protocol [23].	(Dia (29.7 ± 16.2) = DiaExE (41.7 ± 7.2)) < Con (77.5 ± 4.6); DiaExR (60.6 ± 8) and DiaExC (60.6 ± 8) > Dia (29.7 ± 16.2)
	Sperm morphology	Papanicolaou stain according to WHO protocol [23].	Dia (85.2 ± 7.5) < Con (95.4 ± 1.3); DiaExE (88 ± 8.8) = DiaExR (89.1 ± 6.9) = DiaExC (93.4 ± 2.7)
	Sperm motility	WHO protocol [23]. Ten µl of the sperm suspension was located on a microscope slide and covered.	Figure 3
Rosety Rodríguez 2014 [19]	Volume	Semen was collected after 3 days of sexual abstinence (intercourse or masturbation) by manual masturbation into a sterile container on-site and examined within 30 min of ejaculation [23].	Dia (pre, 2.90 ± 0.86) = Dia (post, 2.92 ± 0.90); DiaEx (pre, 2.92 ± 0.90 = DiaEx (post, 3.08 ± 0.86)
	Concentration	Hemacytometer (HauserScientific Inc., Horsham, PA, USA)	Figure 2
	Motility	Computer-aided semen analysis (CASA system, Microptic S.L., Spain)	Figure 3
	Morphology	Hemacytometer (Hauser Scientific Inc., Horsham, PA, USA) and Computer-aided semen analysis (CASA system, MicropticS.L., Spain)	DiaEx (pre, 46.1 ± 4.6) < DiaEx (post, 51.2 ± 5.0) DiaEx (post, 51.2 ± 5.0) > Dia (post, 45.9 ± 4.8)
Murray 1988 [18]	Evaluation of sperm quality before exercise	One single measure comparing diabetic and healthy young males: - Volume: Dia (2.25 ± 0.6); Con (2.98 ± 0.7) - Sperm density: Dia (41.5 ± 10.7); Con (57.4 ± 10.7) - Motility: Dia (46.3 ± 6.1); Con (53.5 ± 4.4)	

Con: control group, Dia: diabetic group, DiaIn: insulin administration group, DiaEx: diabetic and exercise intervention group, DiaInEx: diabetic group with insulin administration and exercise intervention, DiaExE: diabetic and endurance exercise intervention group, DiaExR: diabetic and resistance exercise intervention group, DiaExC: diabetic and combined exercise intervention group, WHO: World Health Organization, HTF: heat transfer fluid.

## Data Availability

Not applicable.

## Appendix A

**Table A1:** Evaluation procedure and results of other parameters measure: histological analysis, hormones, apoptosis markers, and antioxidants.

Study	Variable	Measurement Procedure	Results
Samadian 2019 [16]	Total cell number in 1 mm^2^ of testicular tissue and relative ratios	Immunohistochemical staining (IHC) and light microscope (Olympus-CH-2, Japan)	Con, Ex, diaIn, diaEx and diaInEx > Dia
	Caspase-3 + cell percentage to total cell number in one mm^2^ of testicular tissue	Immunohistochemical staining (IHC) and light microscope (olympus-ch-2, Japan)	Con and Ex < diaIn and diaExIn < diaEx< Dia
	Bax + cell percentage to Bcl-2 + cells in one mm^2^ of testicular tissue	Immunohistochemical staining (IHC) and light microscope (Olympus-CH-2, Japan)	Con and Ex < diaInEx < diaIn and diaEx < Dia
	Negative Tubular Differentiation (TDI) and Spermiogenesis Indices (SPI)	Tissue fixation and staining with hematoxylin and eosin. Tubules containing less than three layers of germinal cells and those with impaired spermiogenesis are negative TDI and SPI, respectively.	TDI: Con, Ex, diaIn and diaInEx < diaEx < Dia SPI: Con, Ex, diaIn and diaInEx < diaEx < Dia
	Relative intensity of Bcl-2/GAPDH	RT-PCR. Mrna isolation, account determined by nanodrop spectrophotometer. Cdna sintetized by Protocol of Fermentas, gmbh, Germany. Rt-PCR by protocol Primers manufactured by Cinna-Gen Co. Final products analysis using PCR Gel software.	Con, Ex, diaIn and diaInEx > diaEx and Dia
	Mean percentages of Bcl-2 + cells	Immunohistochemical staining protocol. Bcl-2 antibody Abcam, catno:ab59348 1:300 was used. Positive IHC reactions were marked as brown stains under a light microscope. The percentage of reacted cells in five microscopic fields was evaluated.	Con, Ex, diaIn and diaInEx > diaEx > Dia
	Relative intensity of Bcl-2 protein/β-Actin	Western blot analyses. Protein evaluation by Lowry method. Bcl-2 antibody Abcam, catno:ab59348 1:300. Blots were visualized using an Enhanced Chemiluminescence Kit (ECL, Thermo scientific) and protein were analyzed using Arash Teb Pishro (ATP) enhanced laser densitometer.	Con, Ex, diaIn and diaInEx > diaEx and Dia
	Relative intensity of Bax/GAPDH	RT-PCR. Mrna isolation, account determined by nanodrop spectrophotometer. Cdna sintetized by Protocol of Fermentas, gmbh, Germany. Rt-PCR Primers designed and manufactured by Cinna-Gen Co. Final products analysis using PCR Gel analyzing software (ATP, Tehran, Iran).	Con and Ex < diaIn, diaEx and diaInEx < Dia
	Mean percentages of Bax + cells	Immunohistochemical staining protocol antibody Bax (Abcam, Cat NO: ab53154,1:500) was used. Positive IHC reactions were marked as brown stains under a light microscope. The percentage of reacted cells in five microscopic fields was evaluated.	Con, Ex and diaInEx < diaIn < diaEx < Dia
	Relative intensity of Bax protein/β-Actin	Western blot analyses. Protein evaluation by Lowry method. Antibody Bax (Abcam, Cat NO: ab53154,1:500). Blots were visualized using an Enhanced Chemiluminescence Kit (ECL, Thermo scientific) and protein were analyzed using Arash Teb Pishro (ATP) enhanced laser densitometer.	Con, Ex, diaIn, diaEx and diaInEx < Dia
	Relative intensity of Caspase-3/GAPDH	RT-PCR. Mrna isolation, account determined by nanodrop spectrophotometer. Cdna sintetized by Protocol of Fermentas, gmbh, Germany. Rt-PCR Primers designed and manufactured by Cinna-Gen Co. Final products analysis using PCR Gel analyzing software (ATP, Tehran, Iran).	Con, Ex < diaIn and diaInEx < diaEx and Dia
	Mean percentages of Caspase-3 + cells	Immunohistochemical staining protocol. Antibody Caspase-3 (Abcam, Cat NO: ab4051, 1:300) was used. Positive IHC reactions were marked as brown stains under a light microscope. The percentage of reacted cells in five microscopic fields was evaluated.	Con and Ex < diaIn and diaInEx < diaEx and Dia
	Relative intensity of Caspase-3 protein/β-Actin	Western blot analyses. Protein evaluation by Lowry method. Antibody Caspase-3 (Abcam, Cat NO: ab4051, 1:300). Blots were visualized using an Enhanced Chemiluminescence Kit (ECL, Thermo scientific) and proteins were analyzed using Arash Teb Pishro (ATP) enhanced laser densitometer.	Con and Ex <diaIn and diaInEx < diaEx and Dia
Samadian 2019 [17]	Testicular weight relative to total body weight	Measure testicular weight relative to total body weight	Con, Ex, diaIn, diaInEx > diaEx, Dia
	Serum level of insulin (ng/mL) 3	Evaluated using special commercial ELISA kits. Insulin (Ray Biotechnology, Cat No: ELR-Insulin)	Con, Ex, diaIn, diaInEx > diaEx > Dia
	Negative Tubular Differentiation (TDI) and Spermiogenesis Indices (SPI)	Tissue fixation and staining with hematoxylin and eosin. Analized using a light microscope Olympus-CH-2, Japan. Tubules containing less than three layers of germinal cells and those with impaired spermiogenesis are negative TDI and SPI, respectively.	TDI: Con, Ex, diaIn, diaInEx < diaEx < Dia SPI: Con Ex, diaIn, diaInEx < diaEx < Dia
	Leydig Cell distribution	Evaluated by TUNEL (terminal deoxynucleotidyl transferase enzyme-mediated dutp nick end labeling) staining kit (In situ cell death detection kit, POD, Roche; Cat, Germany).	Con, Ex, diaIn, diaInEx, diaEx > Dia
	Leydig cell apoptosis	Evaluated by tunel (terminal deoxynucleotidyl transferase enzyme-mediated dutp nick end labeling) staining kit (in situ cell death detection kit, POD, roche; cat, Germany).	Con, Ex < diaIn, diaInEx < diaEx
	Testosterone level	Evaluated using special commercial ELISA kits special kit for rat testosterone (cat no: csb-e05100r).	Con, Ex, diaIn, diaInEx >diaEx >dia
	Testicular Antioxidants level: TAC (total antioxidant activity), SOD, and GPX	Commercial kits: superoxide dismutase (SOD, Zellbio, gmbh, Cat No: ZB-SOD-96A) Glutathione peroxidase (GPX, Zellbio, gmbh, Cat No: ZB-GPX-A96) from Elim-Teb Co. The commercial kit for assessment of tissue total antioxidant capacity (TAC, Cat No: ATAK-105112) was from AYANDEH lab. Co.	TAC: Con, Ex >diaIn, diaInEx > diaEx > Dia SOD: Con, Ex, diaIn, diaInEx > diaEx > Dia GPX: Com, Ex, diaIn, diaInEx > diaEx > Dia
	Intracellular storage of carbohydrates in germinal cell	Periodic acid Schiff (PAS) staining was conducted. The PAS+ cell numbers/mm 2 of tissue were counted and compared between groups.	Con, Ex, diaIn, diaInEx > diaEx > Dia
Parastesh 2019 [20]	Hormonal levels LH	Blood samples were collected by cardiac puncture (5 cc) and centrifuged at 3500 rpm for 10 min and the serum samples were stored at −70 °C (all hormonal analysis). Commercial Kit LH detection (Rat ELISA Kit, Eastbiopharm Cat. No Ck-E90904, China, sensitivity: 0.11 miu/L, Assay range: 0.2–60 miu/L)	Dia (3.9 ± 0.7) < Con (5.6 ± 2.8) Ex (9.8 ± 1.6) > Con, Dia and diaEx (4.7 ± 1)
	Hormonal levels FSH	Commercial Kit FSH (Rat ELISA Kit, Eastbiopharm Cat. No Ck-E30597, China, sensitivity: 0.12 miu/L, Assay range: 0.2–60 miu/L)	Con: 4.4 ± 1 Dia: 3.5 ± 1.1 DiaEx: 5.9 ± 5 Ex: 4.3 ± 1
	Hormonal levels Testosterone	Commercial Kit Testosterone detection (Rat ELISA Kit, Eastbiopharm Cat. No Ck- E90243, China, sensitivity: 0.25 nmol/L, Assay range: 0.5–100 nmol/L)	Dia (4.6 ± 1.6) < Con (6.6 ± 1.8) Ex (7.9 ± 1.7) > Dia and diaEx (5.7 ± 2.3)
	Hormonal levels Adiponectina	Commercial Kit Adiponectin detection (Rat ELISA Kit, Eastbiopharm Cat. No Ck-E30584, China, sensitivity: 0.16 mg/L, Assay range: 0.2–60 mg/L)	Dia (1.6 ± 0.6) and diaEx (3.8 ± 1.1) < Con (5.6 ± 2.2) DiaEx > diaEx (5.3 ± 0.7) > Dia and diaEx
Parastesh 2019 [21]	Hormonal levels LH	Blood samples were collected by cardiac puncture (5 cc) and centrifuged at 3500 rpm for 10 min and the serum samples were stored at −70 °C (all hormonal analysis). Commercial Kit LH detection (Rat ELISA Kit, Eastbiopharm Cat. No Ck-E90904, China, sensitivity: 0.11 miu/L, Assay range: 0.2–60 miu/L)	Dia (3.3 ± 0.4)< Con (5.6 ± 1.6), diaExE (6.7 ± 1.9), diaExR (6.3 ± 1.6) and diaExC (6.3 ± 1.8) > Dia
	Hormonal levels FSH	Commercial Kit FSH (Rat ELISA Kit, Eastbiopharm Cat. No Ck- E30597, China, sensitivity: 0.12 miu/L, Assay range: 0.2–60 miu/L)	Con: 4.1 ± 1.5 DiaExE: 5.8 ± 4.01 DiaExR: 4.4 ± 0.75 DiaExC: 5 ± 4.27 DiaExE (5.8 ± 4.01) > Dia (2.7 ± 0.33)
	Hormonal levels Testosterone	Commercial Kit Testosterone detection (Rat ELISA Kit, Eastbiopharm Cat. No Ck- E90243, China, sensitivity: 0.25 nmol/L, Assay range: 0.5–100 nmol/L	Dia (3.3 ± 0.2) < Con (4.5 ± 0.9), diaExE (4.8 ± 0.9), diaExR (5.2 ± 1.5) and diaExC (5 ± 1.8) > Dia
Rosety-Rodríguez 2014 [19]	TAC (Total Antioxidant capacity)	Seminal plasma was obtained after centrifugation at 3500 rpm for 15 min and it was loaded in the eppendorf tubes (3 mL) and stored at −80 °C until analysis. Colorimetric method using randox commercial kits on the Hitachi 902 autoanalyzer.	DiaEx (pre) (1.41 ± 0.53) < diaEx (post) (1.89 ± 0.46) (*p* < 0.05) > dia (post) (1.36 ± 0.60) (*p* < 0.05) Dia (pre): 1.32 ± 0.58 Within and between group increase in the diabetic exercise group.
	GPX	Seminal plasma was obtained after centrifugation at 3500 rpm for 15 min and it was loaded in the eppendorf tubes (3 mL) and stored at −80 °C until analysis. Subsequent oxidation of NADPH at 240 nm with t-butyl-hydro-peroxide as a substrate. GPX units (u/g protein) were defined as mol NADPH oxidized/g protein.	DiaEx (pre) (4.77 ± 1.66) < diaEx (post) (6.23 ± 1.50) (*p* < 0.05) > dia (post) (4.61 ± 1.55) (*p* < 0.05) Dia (pre): 4.58 ± 1.49 Within and between group increase in the diabetic exercise group.
Murray 1988 [18]	Serum LH	Determined on unextracted urine at pH 7.0 Radioimmunoassays during exercise were performed using the WHO 2nd IRP-HMG reference preparation obtained from Diagnostic Products Corp. (Los Angeles, CA) as Standard.	Dia< Con at rest. It remains lower in Dia during exercise but not in recovery.
	Serum FSH	Radioimmunoassays during exercise were performed using the WHO 2nd IRP-HMG reference preparation obtained from Diagnostic Products Corp. (Los Angeles, CA) as Standard.	Dia < Con at rest, exercise, and recovery. No changes observed during exercise.
	Total testosterone	Established radioimmunoassays.	Dia: Significant increment during exercise and reduction to basal levels in the recovery.

Con: control group, Dia: diabetic group, DiaIn: insulin administration group, DiaEx: diabetic and exercise intervention group, DiaInEx: diabetic group with insulin administration and exercise intervention, DiaExE: diabetic and endurance exercise intervention group, DiaExR: diabetic and resistance exercise intervention group, DiaExC: diabetic and combined exercise intervention group.
